# Quantifying the Interplay Between PRAME Expression and Classical Morphology: A Multivariable Analysis of 954 Suspected Melanocytic Lesions

**DOI:** 10.3390/dermatopathology13030035

**Published:** 2026-08-03

**Authors:** Frank Friedrich Gellrich, Alaska Kistner, Jakob Nikolas Kather, Narmin Ghaffari Laleh, Claudia Günther, Cosima Hufnagel, Sarah Hobelsberger, Jörg Laske, Stefan Beissert, Daniela Aust, Gustavo Baretton, Nick Reidow, Mildred Sergon

**Affiliations:** 1 Department of Dermatology, Faculty of Medicine and University Hospital Carl Gustav Carus, Technical University Dresden, 01304 Dresden, Germany; alaska_carola.kistner@mailbox.tu-dresden.de (A.K.); joerg.laske@ukdd.de (J.L.);; 2 Skin Cancer Center at the University Cancer Center, National Center for Tumor Diseases Dresden, 01304 Dresden, Germany; 3 Institute of Pathology, Faculty of Medicine and University Hospital Carl Gustav Carus, Technical University Dresden, 01304 Dresden, Germany; 4 Else Kroener Fresenius Center for Digital Health, Technical University Dresden, 01304 Dresden, Germany; 5 Department of Medicine I, Faculty of Medicine and University Hospital Carl Gustav Carus, Technical University Dresden, 01304 Dresden, Germany; 6 Department of Dermatology, Faculty of Medicine and University Hospital Tübingen, Eberhard Karls University of Tübingen, 72076 Tübingen, Germany

**Keywords:** melanoma, PRAME, dermatopathology, morphological criteria, differential diagnosis

## Abstract

The integration of immunohistochemical biomarkers like PRAME (Preferentially Expressed Antigen in Melanoma) into the histomorphological assessment of melanocytic lesions is gaining prominence. While PRAME’s diagnostic value is widely recognized, its independent weight compared directly to classical morphology remains underexplored in large, challenging cohorts. This retrospective study quantifies the diagnostic utility of PRAME alongside traditional histomorphology in a high-risk cohort of 954 primary melanocytic lesions (335 nevi, 215 melanomas in situ, 404 invasive melanomas) where PRAME was clinically requested to resolve diagnostic ambiguity. Using Firth’s penalized likelihood regression, a baseline diagnostic model utilizing 13 histomorphological features was established and subsequently integrated with PRAME expression. The pure morphology baseline model demonstrated a high cross-validated area under the curve (AUC) of 0.969. As a standalone marker, PRAME achieved an AUC of 0.895, with a diffuse expression score of 4+ yielding peak specificity (94.3%) and moderate sensitivity (77.9%). Integrating PRAME into the morphological model significantly enhanced diagnostic accuracy (AUC: 0.980; *p* < 0.001), establishing PRAME as a dominant independent predictor of malignancy alongside core features like junctional atypia and upward melanocytes (Odds Ratio 19.08 for Score 4+). Analysis of discordant cases revealed that completely PRAME-negative melanomas were morphologically indistinguishable from typical PRAME-positive melanomas. Conversely, diffusely PRAME-positive (4+) nevi exhibited significantly higher rates of upward migrating melanocytes, constituting a critical diagnostic pitfall. In conclusion, while classic histomorphology remains the indispensable gold standard in dermatopathology, PRAME functions as a highly objective, reproducible tie-breaker. The synergistic integration of PRAME with morphological assessment effectively resolves diagnostic ambiguity and maximizes diagnostic confidence in challenging melanocytic lesions.

## Simple Summary

Diagnosing skin cancer, particularly melanoma, remains one of the most challenging tasks in dermatology because benign moles can closely mimic malignant tumors under the microscope. Recently, a novel laboratory staining marker called PRAME has been introduced to help pathologically differentiate between these entities. However, its accuracy in daily clinical practice, when evaluated alongside traditional tissue features, has not been fully quantified. By analyzing a large real-world archive of over nine hundred cases, this study demonstrates that while this new stain is an exceptionally valuable diagnostic aid, it is not flawless and can occasionally yield false results. Ultimately, the highest diagnostic certainty is achieved not by relying on the stain alone, but by carefully combining its results with classic microscopic tissue examination.

## 1. Introduction

Cutaneous melanoma remains a major global health concern, characterized by rising incidence and an increasing burden of disability-adjusted life years (DALYs) despite stable mortality rates [1,2]. In Germany, a statutory nationwide screening program was introduced in 2008, providing biannual whole-body skin examinations including dermatoscopy for all insured individuals aged 35 and older. Following its implementation, the volume of diagnosed cases has significantly increased [3], with in situ and pT1 lesions rising by 69% [4]. Although essential for early detection [5], these screening efforts have led to a surge in borderline melanocytic lesions that frequently challenge routine histopathological assessment [6]. This clinical landscape highlights the urgent necessity for objective biomarkers like PRAME (Preferentially Expressed Antigen of Melanoma) to improve diagnostic accuracy and resolve ambiguity in morphologically challenging cases.

Histomorphology remains the cornerstone of melanoma diagnosis [7], with the evaluation of the junctional zone being critical for identifying early lesions [7]. A hallmark of malignant transformation is pagetoid melanocytic spread [7,8], molecularly driven by the downregulation of E-cadherin—the so-called cadherin switch [9,10]. While focal upward migration may occur in reactive, inflammatory, or post-operative settings [11], widespread pagetoid spread serves as a strong indicator of malignancy [8]. Additional criteria include the transition from discrete nests to broad confluence [7,8] and the failure of dermal maturation [7]. Although infrequent, dermal mitoses are highly specific for malignancy [8,12]. Finally, host response features such as regression, fibrosis with melanophages, and tumor-infiltrating lymphocytes (TILs) [13,14] provide important diagnostic clues, though they can significantly complicate the definitive assessment of the lesion [12].

Significant interobserver variability in the morphological assessment of borderline lesions—including severely dysplastic nevi, atypical Spitz tumors, traumatized acral lesions, and nevoid melanomas—underlines the utility of PRAME as a diagnostic adjunct [15]. As a cancer-testis antigen (CTA), PRAME is normally silenced in melanocytes through epigenetic methylation [16]. During neoplastic transformation, however, hypomethylation triggers PRAME overexpression [17]. Biologically, PRAME functions as a repressor of the retinoic acid receptor (RAR) signaling pathway, inhibiting cellular differentiation and apoptosis, which in turn promotes aggressive tumor progression [17].

PRAME exhibits high sensitivity in melanoma and melanoma in situ (MIS) while remaining negative in most nevi [15,18,19]. Clones QR005 and EPR20330 are the most validated [20], requiring a red chromogen to effectively distinguish nuclear staining from endogenous melanin. Scoring follows a 0 to 4+ scale; a 4+ score (>75% diffuse staining) has demonstrated sensitivities of 94% in MIS and 79% in invasive melanoma, with fewer than 8% of all melanomas remaining completely negative [16]. While this 4+ cutoff yields peak specificity, Gassenmaier et al. discussed 2+ or 3+ as diagnostic “sweet spots,” providing an optimized balance for clinical routine [21].

However, PRAME is not without its pitfalls. False-negative results occur in specific subtypes, particularly desmoplastic melanoma, which demonstrates diffuse positivity in only 35% of cases and thereby highlights pronounced intra-tumor heterogeneity [16]. Furthermore, expression may be partially or completely lost in rapidly proliferating metastases [22]. A leading explanation for this metastatic loss is active immunoediting within T-cell-rich microenvironments (such as lymph nodes), where cytotoxic T-cells recognize and deplete highly immunogenic PRAME-positive populations, granting a selective survival advantage to PRAME-negative antigen-loss variants [23]. Additionally, while PRAME expression is dynamically linked to promoter hypomethylation, general tumor data indicate that this epigenetic control can be inconsistent, allowing for functional transcriptional variations that further drive phenotypic plasticity [24]. This highlights the indispensable role of morphology in these contexts. Conversely, PRAME positivity can be encountered in certain benign lesions, including the Spitzian spectrum, severely dysplastic nevi, and chronically sun-damaged skin [17,25,26]. Consequently, PRAME should not be interpreted in isolation, particularly when morphological atypia is minimal; in such discordant or highly ambiguous cases, ancillary molecular diagnostics like FISH or NGS remain essential for definitive classification [27].

The primary objective of this retrospective study is to quantify the internal consistency and synergistic interplay between PRAME expression and traditional histomorphological criteria within a high-stakes, real-world diagnostic archive. Rather than framing PRAME as an isolated, detached predictive tool in a vacuum, this analysis characterizes how an objective immunophenotypic surrogate correlates with and calibrates a complex matrix of pre-defined cytomorphological features. By examining this integrated, multi-layered framework, we aim to map the structural harmony between immunophenotype and classical tissue architecture, thereby establishing a reproducible baseline for how objective molecular markers stabilize routine dermatopathological assessment.

## 2. Material and Methods

### 2.1. Patient Selection and Cohort Definition

This retrospective, single-center study, conducted at the Department of Dermatology, University Hospital Carl Gustav Carus, TU Dresden, identified all melanocytic lesions where PRAME immunohistochemistry was requested by the pathologist for diagnostic assessment between 1 January 2020 and 21 January 2025. From an initial retrieval of approximately 1360 cases, non-melanocytic lesions, re-excision specimens, rare entities (e.g., uveal or mucosal melanomas), and metastatic lesions were strictly excluded to focus the analysis on the primary diagnostic challenge. All corresponding hematoxylin and eosin (H&E) and PRAME-stained slides—as well as additional available markers including SOX10, MelanA, HMB45, and Ki-67 (Mib-1)—were digitized using an Aperio AT2 high-volume slide scanner (Leica Biosystems, Wetzlar, Germany) at 40× magnification. Following the exclusion of whole-slide images (WSIs) with significant technical artifacts, one representative WSI was selected for each unique clinical case. In 18 instances where the WSI exhibited only junctional features despite an original diagnosis of a dermal component on other tissue levels, the original reference diagnosis was maintained to preserve the integrity of the gold standard. The final study population consisted of *n* = 954 primary lesions, categorized as melanocytic nevi, melanoma in situ, or invasive melanoma, with complete demographic data and anatomical location available for *n* = 942 cases, which were utilized strictly for descriptive cohort characterization. Clinicopathological subclassifications of benign nevi were extracted directly from the original reference pathology reports, categorizing lesions into dysplastic nevi, Spitz nevi, blue nevi, Reed nevi, combined nevi, and special-site nevi. Diagnoses, including the evaluation of dysplasia, were rendered by the primary pathologists according to standard World Health Organization (WHO) criteria. Due to the retrospective nature of this real-world cohort, a standardized grading of dysplasia could not be systematically retrieved, and no retrospective re-evaluation or sub-grading system was applied. Similarly, melanomas in situ were differentiated into lentigo maligna and classic patterns, while primary prognostic markers (tumor thickness and Clark level) were compiled for all invasive tumors. Crucially, broader clinical attributes such as cumulative solar damage (high-CSD vs. low-CSD) and specific invasive melanoma subtypes (e.g., superficial spreading melanoma, nodular melanoma) were intentionally excluded from the final data matrix due to highly inconsistent, fragmented, and non-standardized reporting within the original historical archive files.

### 2.2. Reference Standard and Study Annotation

The reference diagnosis was obtained from the original clinical pathology reports, which were authored by board-certified dermatopathologists. All malignant diagnoses (melanoma in situ and invasive melanoma) followed a mandatory institutional “four-eyes” principle (second-opinion review), with rare borderline cases resolved through interdisciplinary consensus meetings. For this study, a single, independent investigator—blinded to both the original diagnosis and PRAME status—retrospectively annotated the 18 morphological criteria and PRAME score. This specific panel of 18 criteria was custom-devised by the authors to represent the core architectural and cytomorphological parameters established in classical dermatopathological literature [7,8,12] without undergoing prior, independent formal validation as a locked multi-variable instrument. This broad, expert-consensus-driven matrix was selected to ensure a comprehensive evaluation of all standard diagnostic variables during the retrospective data annotation. During the morphological annotation, intraepidermal melanocytic migration was evaluated hierarchically. The overarching category “upward melanocytes” encompassed any migration above the basal layer, ranging from isolated single cells to dense infiltration. During evaluation, this feature was highlighted by lineage-specific immunohistochemistry (such as SOX10 or MelanA), which was available in most archive cases and assessed concurrently with the conventional H&E sections. Within this spectrum, “wide pagetoid spread” was recorded as a specific subcategory, strictly reserved for advanced, multi-layered, and partially nested infiltration of the upper epidermis. Due to the blinded design, the feature ‘regression’ was recorded as a purely morphological pattern (horizontal fibrosis and neocapillarization), encompassing both spontaneous biological regression and potential post-interventional scarring. To ensure a mathematically complete dataset required for multivariable regression and to prevent selection bias, all 18 histomorphological criteria were systematically annotated for every specimen, with absent or non-evaluable features strictly recorded as a binary “no” (not present).

### 2.3. Immunohistochemistry and PRAME Scoring

Staining was performed on 4 μm FFPE sections using the PRAME antibody (clone QR005, Quartett, 1:100 dilution) on an automated Ventana BenchMark ULTRA platform (Roche Diagnostics, Tucson, AZ, USA) with the UltraView Fast Red detection system to avoid confusion with melanin. PRAME expression was scored using a five-tier system (0 to 4+) based on the percentage of positive tumor nuclei. Any discernible nuclear staining, regardless of intensity, was considered positive if distinguishable from background noise. A full definition of the scoring and the morphological catalog is provided in the (Supplementary Materials).

### 2.4. Statistical Analysis

Analyses were performed in R (v4.4.3) and RStudio (2026.1.2.418). We employed Firth’s penalized likelihood regression (logistf) to identify independent predictors and account for quasi-complete separation. To optimize the model’s diagnostic focus, specific features were excluded from multivariable analysis based on clinico-pathological rationale: structural parameters (junctional/dermal involvement, subcutaneous infiltration) were omitted as they relate to lesion categorization rather than malignancy prediction, and inflammatory markers (dense infiltrate, tumor-infiltrating lymphocytes) were excluded as they represent host response rather than primary cytomorphological criteria.

To ensure robust performance estimates and prevent overfitting, all receiver operating characteristic (ROC) curves and corresponding areas under the curve (AUC) were generated using 10-fold cross-validation. The primary multivariable model (*n* = 954) integrated PRAME with 13 morphological features. Direct statistical comparison between the morphology baseline and combined models was performed using the DeLong test. To control for Type I errors, *p*-values were adjusted using the Benjamini–Hochberg False Discovery Rate (FDR) method, with *p* < 0.05 considered significant. A complete-case analysis was ensured by the initial exclusion of non-evaluable cases.

## 3. Results

### 3.1. Cohort Characteristics and Demographic Distribution

The final study population comprised a total of 954 primary melanocytic lesions, categorized into 335 nevi (35.1%), 215 melanomas in situ (MIS; 22.5%), and 404 invasive melanomas (42.3%; Table 1). The median age of the entire cohort was 64 years, with significant differences between diagnostic groups (*p* < 0.001); patients with nevi were notably younger (median 40 years) compared to those with MIS (median 74 years) or invasive melanomas (median 70 years). Males represented 55% (*n* = 522) of the total cohort, with a significantly higher proportion in malignant diagnoses (MIS: 56%; invasive melanoma: 63%) compared to the nevus group (44%; *p* < 0.001). Regarding anatomical localization (*n* = 942 documented cases), the trunk was the most frequent site overall (45%), followed by the head and neck area (25%), the lower extremities (19%), and the upper extremities (12%).

To resolve diagnostic granularity while maintaining analytical focus, detailed clinicopathological subclassifications were evaluated within each major diagnostic category (Table 1). Within the nevus cohort (*n* = 335), dysplastic nevi accounted for 47.2% (*n* = 158) of cases, whereas the frequencies of the remaining specific benign entities (including Spitz, blue, and Reed nevi) are detailed directly in Table 1. For the invasive melanoma subset (*n* = 404), the median tumor thickness was 0.90 mm (IQR: 0.50–1.90 mm). The architectural distribution across Clark levels revealed 24% at level II (*n* = 98), 41% at level III (*n* = 164), 22% at level IV (*n* = 89), and 5.2% at level V (*n* = 21), with 7.9% (*n* = 32) of invasive cases lacking historical documentation of the Clark level within the archive.

To ensure high objectivity and reproducibility, the histological evaluation was strictly limited to 18 pre-defined morphological criteria, which were assessed retrospectively and in a fully blinded manner. The evaluation documented a high prevalence of morphological features typically associated with diagnostic challenges (Table 2): distinct junctional atypia was present in 60% of all cases, and nest confluence was observed in 63% of lesions. The presence of upward melanocytes as an overarching feature was recorded in 70% of the total cohort, with the advanced subcategory of wide pagetoid spread being documented in 42% of cases (Table 2). Within the inflammatory context, a dense lymphocytic infiltrate was found in 39% of cases, while tumor-infiltrating lymphocytes (TILs) were documented in 25%. High rates of cytological atypia and structural irregularities were observed across the entire dataset, including the nevus group. This morphological profile reflects the retrospective nature of the cohort, which consisted of cases where PRAME immunohistochemistry had been requested. All *p*-values regarding clinical and morphological data were adjusted using the Benjamini–Hochberg (FDR-BH) method to account for multiple testing.

### 3.2. Diagnostic Performance of Morphology (Baseline Model)

**Figure 1:**
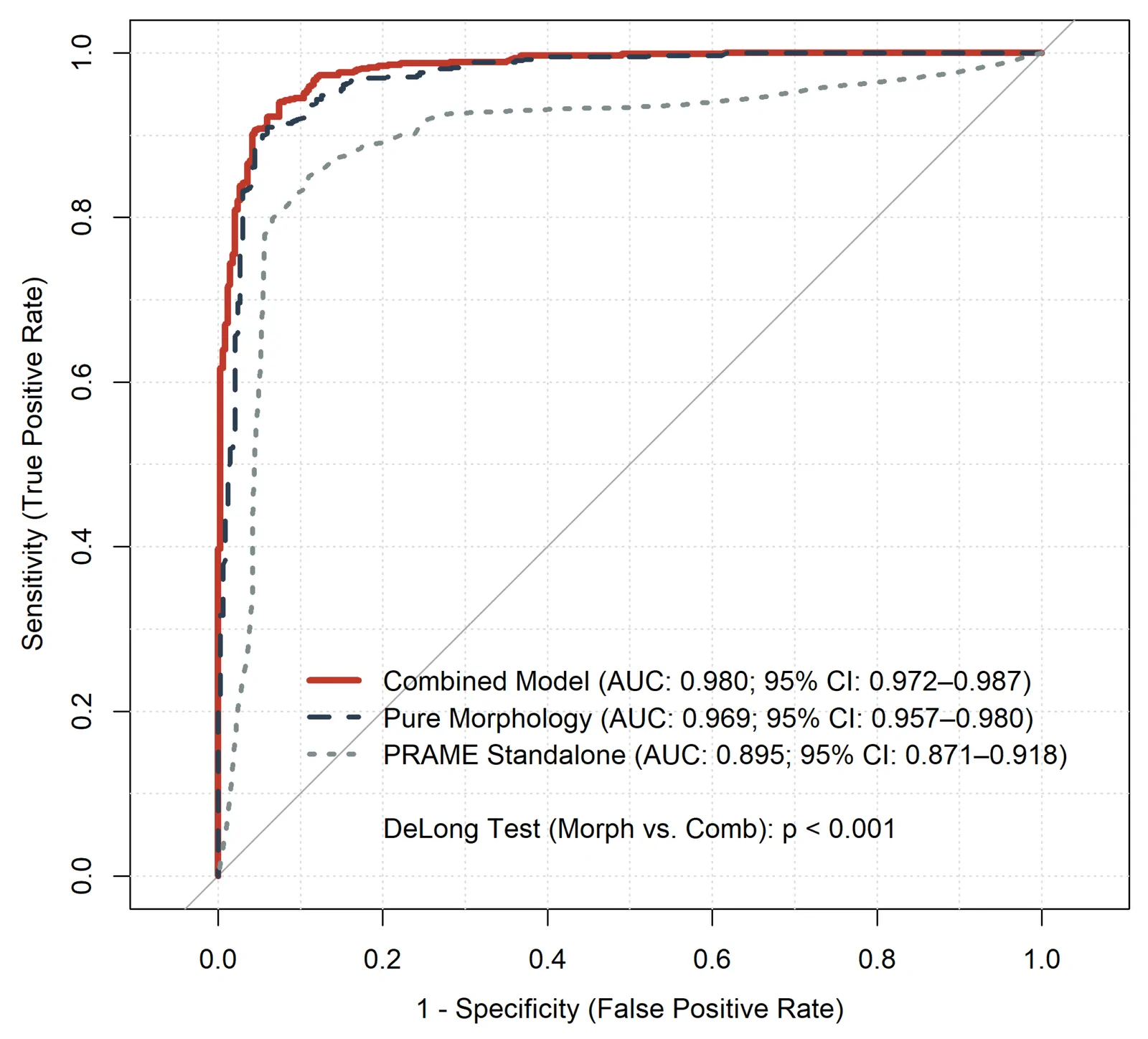
ROC curve analysis of diagnostic models. Comparison of performance for the combined model (solid red line), the pure morphology baseline (long-dashed black line), and PRAME as a standalone marker (short-dashed grey line) based on the total cohort (*n* = 954). Abbreviations: AUC, area under the curve; CI, confidence interval.

**Figure 2:**
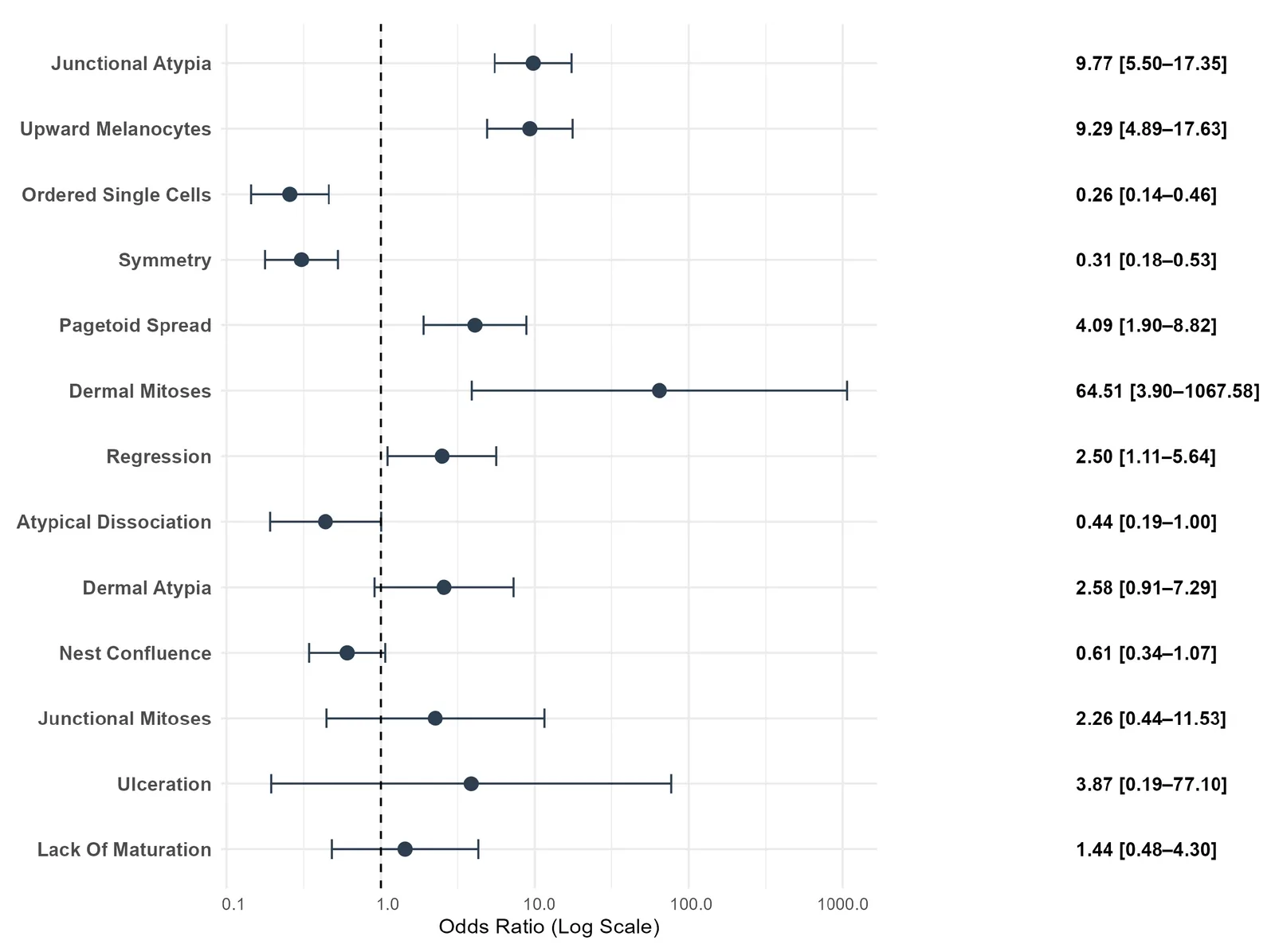
Forest plot of histomorphological features. Visualization of the diagnostic impact of the thirteen selected features. Odds Ratios are presented on a logarithmic scale, with corresponding numerical point estimates and 95% Confidence Intervals listed on the right side of the plot.

To establish a diagnostic benchmark without the aid of the PRAME score, a baseline model was constructed using 13 selected histomorphological features. Utilizing Firth’s penalized likelihood regression on the total cohort (*n* = 954), this pure morphology model achieved a high cross-validated area under the curve (AUC) of 0.969 (Figure 1). The relative diagnostic weights and statistical robustness of these features were visualized in a forest plot (Figure 2), with detailed performance metrics provided in the supplementary materials (Supplementary Table S1).

The most statistically robust independent predictors of malignancy were distinct junctional atypia (OR 9.77; 95% CI: 5.50–17.35; *p* < 0.001) and the presence of upward melanocytes (OR 9.29; 95% CI: 4.89–17.63; *p* < 0.001). A wide pagetoid spread also served as a significant independent indicator of malignancy as a specific subcategory (OR 4.09; 95% CI: 1.90–8.82; *p* = 0.001). While dermal mitoses exhibited the highest overall effect size (OR 64.51; 95% CI: 3.90–1067.58; *p* = 0.008), the wide confidence interval reflects their high specificity coupled with a relatively low frequency within the cohort.

Conversely, morphological symmetry (OR 0.31; 95% CI: 0.18–0.53; *p* < 0.001) and an ordered single-cell pattern in the dermis (OR 0.26; 95% CI: 0.14–0.46; *p* < 0.001) were identified as the strongest indicators of a benign diagnosis. Other parameters, such as ulceration and nest confluence, did not reach independent significance in this multivariable context.

### 3.3. Diagnostic Performance of PRAME (Univariable)

Nuclear PRAME expression, which was likewise evaluated retrospectively and in a blinded manner, demonstrated highly significant differences across the diagnostic categories (*p* < 0.001; Table 1). Within the benign group (*n* = 335 nevi), the majority of lesions (72.8%) were completely negative (score 0), while diffuse positivity (score 4+) was restricted to 5.7% of cases. Conversely, malignant lesions were characterized by high-level PRAME expression. Diffuse nuclear staining (score 4+) was present in 75.8% of melanomas in situ (*n* = 215) and 79.0% of invasive melanomas (*n* = 404). Complete absence of staining (score 0) was observed in only 6.5% of MIS and 7.9% of invasive melanomas.

As a standalone diagnostic tool for differentiating malignant from benign lesions, PRAME immunohistochemistry achieved an Area Under the Curve (AUC) of 0.895 (95% CI: 0.871–0.918; Figure 1). The diagnostic performance metrics shifted according to the selected positivity threshold (Table 3). At the lowest threshold (score ≥1+), the marker reached its highest sensitivity of 92.6% (95% CI: 90.2–94.5%) with a corresponding specificity of 72.8% (95% CI: 67.7–77.5%). Utilizing a threshold of diffuse expression (score ≥4+) maximized specificity at 94.3% (95% CI: 91.3–96.6%), while sensitivity declined to 77.9% (95% CI: 74.4–81.1%). The peak diagnostic accuracy in this cohort was identified at the intermediate cut-off levels, specifically at ≥2+ (86.5%) and ≥3+ (86.4%).

### 3.4. Multivariable Model and Statistical Comparison

To evaluate the synergistic effect of combining histomorphology with immunohistochemistry, PRAME expression was integrated into the morphological baseline model. This combined multivariable model demonstrated superior diagnostic performance, achieving a cross-validated area under the curve (AUC) of 0.980 (Figure 1). This represents a marked improvement over the morphology-only approach, which yielded an AUC of 0.969. A direct statistical comparison of the two ROC curves using the DeLong test confirmed that the addition of PRAME significantly enhanced discriminatory power (*p* < 0.001; Supplementary Table S2).

**Figure 3:**
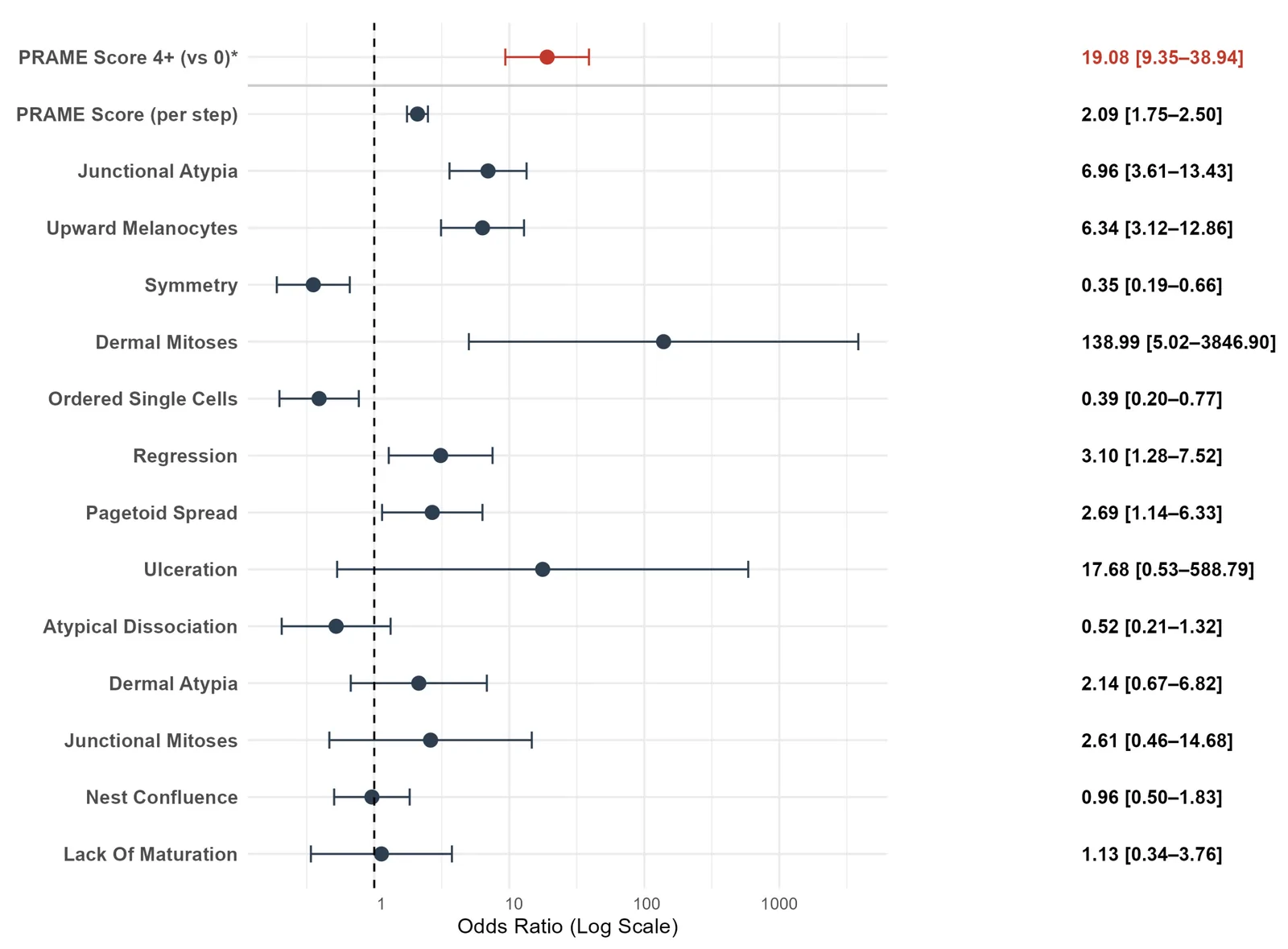
Forest plot of the combined diagnostic model including PRAME. This visualization illustrates the diagnostic impact of histomorphological features integrated with PRAME expression. Odds Ratios are presented on a logarithmic scale, with corresponding numerical point estimates and 95% Confidence Intervals provided on the right side of the plot. The red entry marked with an asterisk (*) denotes the specifically calculated comparison between a PRAME score of 4+ and a score of 0.

In the multivariable ranking, PRAME emerged as a dominant independent predictor of malignancy (Figure 3). When analyzed as a stepwise variable, each incremental increase in the PRAME score significantly raised the odds of a melanoma diagnosis (OR 2.09; 95% CI: 1.75–2.50; *p* < 0.001). Specifically, diffuse PRAME expression (score 4+) was associated with a 19-fold increase in the odds of malignancy compared to completely negative lesions (OR 19.08; 95% CI: 9.35–38.94).

The combined model also retained the most robust morphological predictors identified in the baseline analysis. Junctional atypia (OR 6.96; 95% CI: 3.61–13.43; *p* < 0.001) and the presence of upward melanocytes (OR 6.34; 95% CI: 3.12–12.86; *p* < 0.001) remained highly significant independent factors. While dermal mitoses demonstrated the highest point estimate for the odds ratio (OR 138.99; *p* = 0.012), the wide confidence interval (95% CI: 5.02–3846.90) indicates high specificity but limited numerical stability due to the rarity of this feature. Model stability was further confirmed through 10-fold cross-validation, which showed consistent performance across the dataset and underscored the reliability of the combined diagnostic approach.

### 3.5. Analysis of Discordant Cases and Diagnostic Pitfalls

A systematic comparison was performed between extreme diagnostic outliers—completely PRAME-negative melanomas (Score 0) and diffusely PRAME-positive nevi (Score 4+)—and their respective concordant counterparts. Across over 60 statistical comparisons involving clinical demographics, anatomical localization, and histomorphological criteria, these outliers demonstrated a high degree of homogeneity with typical cases (Supplementary Table S2).

For both melanoma in situ and invasive melanoma, no significant morphological differences were identified between Score 0 and Score 1+ to 4+ cases. Primary malignancy criteria including junctional atypia, architectural disorder, and mitotic activity occurred at comparable frequencies regardless of PRAME status. Clinical baseline characteristics such as age, sex, and anatomical localization demonstrated no significant correlation with PRAME expression in either cohort, with all *p*-values exceeding 0.05.

Within the nevus group, Score 4+ lesions exhibited three significant deviations compared to Score 0–3 cases. A dermal component was less frequently present (32% vs. 82%; *p* < 0.001), and a regular maturation pattern (ordered single cells) was observed less often (11% vs. 65%; *p* < 0.001). Because the total nevus cohort included junctional, compound, and dermal subtypes, and these features are structurally dependent on the presence of a dermal component, these findings are subject to the distribution of nevus subtypes within the small outlier subgroup (*n* = 19). Independently, Score 4+ nevi demonstrated a significantly higher frequency of upward migrating melanocytes compared to PRAME 0 to 3+ nevi (58% vs. 27%; *p* = 0.036). Within this specific high-risk outlier subgroup, the original reference diagnoses comprised 12 dysplastic nevi, three Spitz nevi, three unspecified common nevi, and one special-site nevus. No other clinical or morphological parameters showed significant differences between the two groups.

## 4. Discussion

To bridge the gap between mathematical modeling and daily microscopic practice, the data interpretation follows a specific clinical-statistical logic. The cross-validated logistic regression used here functions primarily as a statistical “stress test” to identify those features that contribute most consistently to a diagnosis within the cohort. In this process, the model weights the individual criteria mathematically to achieve the highest theoretical diagnostic accuracy. The resulting AUC values thus describe a performance ceiling under mathematically optimal weighting, which may exceed the intuitive weighting used in daily clinical routine.

In this context, the Odds Ratios (OR) represent the independent added value of a feature within the overall “diagnostic team”. Since the model accounts for multicollinearity, a highly relevant feature may show a smaller OR if its information is already statistically covered by another closely related criterion (e.g., upward melanocytes vs. pagetoid spread). The OR thus describes the specific, independent contribution of a factor. Finally, the *p*-values serve as a filter for the reliability of these contributions, separating actual diagnostic drivers from random effects. For this reason, the results were consistently sorted by their level of significance to highlight the most reliable parameters for histopathological routine.

### 4.1. Summary of Main Findings

This study highlights the powerful synergy that occurs when PRAME immunohistochemistry is systematically combined with classical morphology, demonstrating that PRAME carries the strongest diagnostic weight for malignancy within this challenging cohort [7,16]. Rather than treating PRAME as an isolated marker, our multivariable analysis reflects the deep internal alignment between objective immunophenotypic signals and tissue architecture, showing how closely both components interlock when resolving borderline uncertainty in routine practice [11,22]. Within this diagnostic team, PRAME functions as a leading anchor whose strong influence works in close harmony alongside core morphological features like junctional atypia [28].

### 4.2. PRAME as a Leading Diagnostic Predictor

In alignment with seminal studies by Lezcano et al. [16,19] and Gassenmaier et al. [25], our results confirm PRAME as a superior diagnostic adjunct. The strong diagnostic weight observed in our analysis is primarily a result of the meticulous, blinded histological correlation and the high-risk nature of this specific borderline cohort. However, as noted in our methodology, these statistical measures are not absolute constants; they are inherently dependent on cohort composition and the specific variables included in the framework, thus primarily reflecting the relative diagnostic strength of the marker within this study population.

Diagnostic performance metrics (Table 3) further highlight this clinical utility. While a PRAME score of ≥4+ achieves the highest specificity (94.3%) for melanoma, it is associated with a moderate sensitivity (77.9%). This finding is highly comparable to the 83.2% sensitivity for primary melanomas reported by Lezcano et al. [16], with the slight variance likely reflecting our specific cohort of morphologically challenging cases. The diagnostic ‘sweet spot’ for balanced accuracy was found at the ≥2+ (86.5%) and ≥3+ (86.4%) cutoffs. This aligns with recent observations by Noyan Mod et al., who identified the 50% threshold (≥3+) as an optimized balance for clinical routine, providing significantly higher sensitivity while maintaining robust specificity [21].

### 4.3. The Synergistic Value of PRAME as a Diagnostic Tie-Breaker

Despite the statistical power of PRAME, classic histomorphology remains the undisputed gold standard and the primary diagnostic framework [7,12]. Indeed, standalone PRAME expression (AUC: 0.895) is notably inferior to the performance of pure morphological assessment (AUC: 0.969), reaffirming that the diagnosis of melanocytic lesions cannot be reduced to a single immunohistochemical marker. However, the addition of PRAME to the morphological model provides a significant synergistic boost, reaching an AUC of 0.985. It must be noted that these high AUC values represent a mathematical ideal where thirteen morphological criteria are weighted with perfect precision—a standard unattainable in the daily routine of intuitive heuristics. Consequently, the true clinical merit of PRAME lies not just in a marginal statistical gain, but in its role as a highly objective, reproducible tie-breaker [15]. In diagnostic “gray zones” where subtle morphological signals may be ambiguous or conflicting, PRAME serves as a crucial anchor, reducing subjective variability and providing a level of diagnostic confidence that exceeds the sum of individual morphological findings [22].

A critical finding of this study is the morphological and clinical “invisibility” of the PRAME status in outlier cases. A direct comparison of PRAME-negative melanomas (Score 0) with their PRAME-positive counterparts revealed no significant differences in classic histomorphology or clinical demographics such as age and sex. Malignancy criteria like distinct junctional atypia and mitotic activity were present at near-identical frequencies regardless of the PRAME score. This underscores that PRAME provides independent biological information [17,18], but it also serves as a warning: PRAME status cannot be anticipated by specific phenotypic or clinical patterns. Consequently, a negative PRAME result must never be trusted blindly and should never override a malignant morphology [27]. Conversely, PRAME 4+ nevi represent a dangerous diagnostic pitfall, as they demonstrated a significantly higher rate of upward migrating melanocytes compared to PRAME 0 to 3+ nevi (58% vs. 27%; *p* = 0.036). Since upward migration is a strong predictor of malignancy in the model—and can be subtle enough to require IHC (e.g., SOX10) for reliable identification [9]—the combination of upward melanocytes and diffuse PRAME positivity represents a high-risk constellation where pathologists must exercise extreme caution [26].

### 4.4. Clinical Implications: Moving Toward an Integrated Diagnostic Model

The findings of this study advocate for a fundamental shift in the diagnostic approach to melanocytic lesions, moving away from a competitive “morphology versus immunohistochemistry” paradigm toward a synergistic integrated model [28]. Historically, immunohistochemistry has often been used as a secondary “safety net” when morphology fails. However, our data suggest that the highest level of diagnostic security is achieved when both are treated as co-dependent components of a single integrated framework.

While traditional histomorphology remains the indispensable starting point, the inherent subjectivity and the variable weighting of its criteria often create diagnostic “gray zones” that are difficult to navigate by intuition alone [11]. By integrating PRAME as an objective, weighted parameter, the diagnostic process gains a stabilizing anchor. This integrated approach effectively mimics the multi-layered reasoning of expert dermatopathologists but adds a layer of mathematical consistency. Ultimately, moving toward such a model-based approach ensures that complex morphological findings are validated by objective molecular surrogates, leading to more reproducible diagnoses and, consequently, more standardized patient management [5].

## 5. Limitations

Several limitations warrant consideration. First, as a monocentric, retrospective analysis without external validation, the model’s performance on independent cohorts remains to be established. Second, while institutional reference diagnoses for malignant cases historically relied on a double-observer consensus, the retrospective re-evaluation of study variables was conducted by a single investigator. Although fully blinded to mitigate review bias, potential intra-observer variability and holistic cognitive bias—where an intuitive impression unconsciously influences individual criteria scoring—cannot be entirely excluded. Third, an inherent diagnostic loop exists because historical reference diagnoses incorporated both morphology and PRAME. However, because all morphological criteria and PRAME scores were independently re-evaluated in a blinded manner, this bias was systematically controlled. Consequently, rather than validating against genetic gold standards like NGS or FISH, this study strictly quantifies the internal phenotypic consistency between immunophenotypic data and classical tissue architecture. However, because no independent molecular reference standard was available and the original histopathological diagnosis necessarily served as the reference standard, a small proportion of biologically misclassified lesions cannot be entirely excluded. Fourth, inconsistent historical reporting precluded the multivariable integration of specific melanoma variants and chronic solar damage scores. Finally, the absence of longitudinal outcome data prevents the correlation of PRAME expression with clinical survival metrics, appropriately limiting the study’s scope to the diagnostic calibration at the time of initial assessment.

## 6. Conclusions

In conclusion, PRAME immunohistochemistry serves as a highly valuable diagnostic aid for challenging melanocytic lesions. However, the occurrence of false-positive and false-negative cases underscores that classic histomorphology remains the indispensable foundation of dermatopathology. Ultimately, the highest level of diagnostic confidence is reached through the cross-modality consistency between architectural morphology and objective immunophenotypic evidence.

## Figures and Tables

**Table 1:** Cohort Characteristics and Clinicopathological Subtypes (*n* = 954). Clinical baseline and PRAME expression scores remained unadjusted. Clinicopathological subclassifications are descriptive and restricted to their respective entities. Statistical significance was evaluated using the Kruskal–Wallis test for continuous data (age) and Pearson’s chi-squared test with Monte Carlo simulated *p*-values for categorical variables (sex, body site, and PRAME score).

Characteristic	Total Cohort (N = 954) ^1^	Nevus N = 335 ^1^	Melanoma In Situ N = 215 ^1^	Invasive Melanoma N = 404 ^1^	*p*-Value (adj.)
**Demographics & Clinical Background**					
**Age (Years)**	64 (44, 77)	40 (25, 60)	74 (61, 81)	70 (58, 81)	<0.001
**Sex**					<0.001
Male	522/954 (55%)	147/335 (44%)	121/215 (56%)	254/404 (63%)	<0.001
Female	432/954 (45%)	188/335 (56%)	94/215 (44%)	150/404 (37%)	<0.001
**Body Site**					<0.001
Head/Neck	234/942 (25%)	61/326 (19%)	104/215 (48%)	69/401 (17%)	<0.001
Trunk	421/942 (45%)	170/326 (52%)	64/215 (30%)	187/401 (47%)	<0.001
Upper Extremity	111/942 (12%)	20/326 (6.1%)	27/215 (13%)	64/401 (16%)	<0.001
Lower Extremity	176/942 (19%)	75/326 (23%)	20/215 (9.3%)	81/401 (20%)	<0.001
**Histopathology & Clinicopathological Subtypes**					
**PRAME Score (0** **–** **4)**					<0.001
0	290/954 (30%)	244/335 (73%)	14/215 (6.5%)	32/404 (7.9%)	<0.001
1	56/954 (5.9%)	32/335 (9.6%)	9/215 (4.2%)	15/404 (3.7%)	<0.001
2	45/954 (4.7%)	22/335 (6.6%)	9/215 (4.2%)	14/404 (3.5%)	<0.001
3	62/954 (6.5%)	18/335 (5.4%)	20/215 (9.3%)	24/404 (5.9%)	<0.001
4	501/954 (53%)	19/335 (5.7%)	163/215 (76%)	319/404 (79%)	<0.001
**Nevus Subclassification (overlapping)**					
Blue Nevus		17/335 (5.1%)			
Spitz Nevus		22/335 (6.6%)			
Combined Nevus		4/335 (1.2%)			
Reed Nevus		3/335 (0.9%)			
Special-site Nevus		12/335 (3.6%)			
Dysplastic Nevus		158/335 (47.2%)			
**Common Nevus (Unspecified)**		119/335 (35.5%)			
**Invasive Melanoma Parameters**					
Tumor Thickness (mm), Median [IQR]				0.90 (0.50, 1.90)	
**Clark Level (II** **–** **V)**					
II				98/404 (24%)	
III				164/404 (41%)	
IV				89/404 (22%)	
V				21/404 (5.2%)	
Not documented (NA)				32/404 (7.9%)	

^1^ Median (Q1, Q3); n/N (%).

**Table 2:** Distribution of Pre-defined Histomorphological Criteria across Diagnostic Categories (*n* = 954). *p*-values for morphological data were independently FDR-BH adjusted within their hypothesis family.

Morphological Criteria	Total Cohort (N = 954) ^1^	Nevus N = 335 ^1^	Melanoma In Situ N = 215 ^1^	Invasive Melanoma N = 404 ^1^	*p*-Value (adj.)
Junctional component present	867/954 (91%)	269/335 (80%)	215/215 (100%)	383/404 (95%)	<0.001
Dermal component present	687/954 (72%)	264/335 (79%)	41/215 (19%)	382/404 (95%)	<0.001
Symmetry present	323/954 (34%)	219/335 (65%)	65/215 (30%)	39/404 (9.7%)	<0.001
Upward melanocytes	668/954 (70%)	95/335 (28%)	205/215 (95%)	368/404 (91%)	<0.001
Pagetoid spread	396/954 (42%)	10/335 (3.0%)	102/215 (47%)	284/404 (70%)	<0.001
Nest confluence	600/954 (63%)	174/335 (52%)	127/215 (59%)	299/404 (74%)	<0.001
Distinct junctional atypia	573/954 (60%)	33/335 (9.9%)	169/215 (79%)	371/404 (92%)	<0.001
Junctional mitoses present	158/954 (17%)	1/335 (0.3%)	30/215 (14%)	127/404 (31%)	<0.001
Lack of maturation	298/954 (31%)	15/335 (4.5%)	2/215 (0.9%)	281/404 (70%)	<0.001
Distinct dermal atypia	289/954 (30%)	14/335 (4.2%)	2/215 (0.9%)	273/404 (68%)	<0.001
Dermal mitoses present	124/954 (13%)	0/335 (0%)	0/215 (0%)	124/404 (31%)	<0.001
Ordered single cells (benign pattern)	261/954 (27%)	206/335 (61%)	27/215 (13%) ^2^	28/404 (6.9%)	<0.001
Atypical dissociation (malignant pattern)	268/954 (28%)	32/335 (9.6%)	2/215 (0.9%)	234/404 (58%)	<0.001
Regression	140/954 (15%)	18/335 (5.4%)	35/215 (16%)	87/404 (22%)	<0.001
Dense lymphocytic infiltrate	369/954 (39%)	61/335 (18%)	68/215 (32%)	240/404 (59%)	<0.001
Tumor-infiltrating lymphocytes (TILs)	237/954 (25%)	30/335 (9.0%)	25/215 (12%)	182/404 (45%)	<0.001
Ulceration	46/954 (4.8%)	0/335 (0%)	1/215 (0.5%)	45/404 (11%)	<0.001
Subcutis infiltration	23/954 (2.4%)	5/335 (1.5%)	0/215 (0%)	18/404 (4.5%)	0.001

^1^ n/N (%). ^2^ Represents cases of melanoma in situ arising in association with a pre-existing conventional compound or dermal nevus.

**Table 3:** Diagnostic Accuracy of PRAME at Different Cutoff Levels (Malignant vs. Benign). Performance metrics comparing Malignant (Melanoma in situ and Invasive Melanoma) vs. Benign (Nevus) lesions. 95% Confidence Intervals (CI) are provided in brackets.

Cutoff Level	TP	FP	FN	TN	Sensitivity	Specificity	Accuracy
PRAME Score ≥ 1+	573	91	46	244	92.6% [90.2–94.5%]	72.8% [67.7–77.5%]	85.6%
PRAME Score ≥ 2+	549	59	70	276	88.7% [85.9–91.1%]	82.4% [77.9–86.3%]	86.5%
PRAME Score ≥ 3+	526	37	93	298	85% [81.9–87.7%]	89% [85.1–92.1%]	86.4%
PRAME Score ≥ 4+	482	19	137	316	77.9% [74.4–81.1%]	94.3% [91.3–96.6%]	83.6%

## Data Availability

To ensure the reproducibility of the results, a reduced dataset has been generated that is fully anonymized and contains only the criteria presented in this article. This dataset is included as a Supplementary Information file.

## Supplementary Materials

The following supporting information can be downloaded at: https://www.mdpi.com/article/10.3390/dermatopathology13030035/s1, Table S1: Diagnostic Performance: Pure Morphology (Baseline Model); Table S2: Intra-Entity Comparison of PRAME Outliers.
