# Supplementary material for: Quantifying the Interplay Between PRAME Expression and Classical Morphology: A Multivariable Analysis of 954 Suspected Melanocytic Lesions
Source: Dermatopathology (Basel). 2026 Aug 3;13(3):35. doi: 10.3390/dermatopathology13030035 (PMC13510435; doi:10.3390/dermatopathology13030035)
Supplement: Supplementary file 1 [file dermatopathology-13-00035-s001.zip › Supplementary Tables.pdf]

Supplementary Tables

Supplementary Table S1: Diagnostic Performance: Pure Morphology (Baseline Model)

This multivariable model (n = 954) includes 13 cytomorphological features. P-values were adjusted using the Benjamini-Hochberg (FDR) method. The cross-validated AUC is 0.969 (95% CI: 0.957-0.98).

**Morphological Feature**	**Odds Ratio [95% CI]**	**p-value (FDR)**
Junctional Atypia	9.77 [5.50 - 17.35]	< 0.001
Upward Melanocytes	9.29 [4.89 - 17.63]	< 0.001
Ordered Single Cells	0.26 [0.14 - 0.46]	< 0.001
Symmetry	0.31 [0.18 - 0.53]	< 0.001
Pagetoid Spread	4.09 [1.90 - 8.82]	< 0.001
Dermal Mitoses	64.51 [3.90 - 1067.58]	0.008
Regression	2.50 [1.11 - 5.64]	0.050
Atypical Dissociation	0.44 [0.19 - 1.00]	0.083
Dermal Atypia	2.58 [0.91 - 7.29]	0.108
Nest Confluence	0.61 [0.34 - 1.07]	0.112
Junctional Mitoses	2.26 [0.44 - 11.53]	0.386
Ulceration	3.87 [0.19 - 77.10]	0.407
Lack Of Maturation	1.44 [0.48 - 4.30]	0.513

Supplementary Table S2: Intra-Entity Comparison of PRAME Outliers

Significant adjusted p-values (<0.05) are highlighted in bold.

	Nevus			Melanoma in situ			Invasive Melanoma		
**Characteristic**	**Score 0-3** (n=316)[1]	**Score 4** (n=19)[1]	**p (adj.)**[2]	**Score 0** (n=14)[1]	**Score 1-4** (n=201)[1]	**p (adj.)**[2]	**Score 0** (n=32)[1]	**Score 1-4** (n=372)[1]	**p (adj.)**[2]
**Age (Years)**	40 (25, 58)	46 (32, 69)	0.769	69 (42, 79)	74 (61, 81)	0.301	69 (55, 79)	70 (58, 81)	0.342
**Sex**			0.814			>0.999			0.342
Male	138 (44%)	9 (47%)	0.814	8 (57%)	113 (56%)	>0.999	23 (72%)	231 (62%)	0.342
Female	178 (56%)	10 (53%)	0.814	6 (43%)	88 (44%)	>0.999	9 (28%)	141 (38%)	0.342
**Body Site**			0.769			0.301			0.342
Head/Neck	59 (19%)	2 (11%)	0.769	4 (29%)	100 (50%)	0.301	3 (9.4%)	66 (18%)	0.342
Trunk	161 (52%)	9 (47%)	0.769	7 (50%)	57 (28%)	0.301	20 (63%)	167 (45%)	0.342
Upper Extr.	18 (5.9%)	2 (11%)	0.769	1 (7.1%)	26 (13%)	0.301	3 (9.4%)	61 (17%)	0.342
Lower Extr.	69 (22%)	6 (32%)	0.769	2 (14%)	18 (9.0%)	0.301	6 (19%)	75 (20%)	0.342
**Junctional component present**	250 (79%)	19 (100%)	0.127	14 (100%)	201 (100%)		29 (91%)	354 (95%)	0.954
**Dermal component present**	258 (82%)	6 (32%)	**<0.001**	3 (21%)	38 (19%)	>0.999	30 (94%)	352 (95%)	0.955
**Symmetry present**	206 (65%)	13 (68%)	>0.999	6 (43%)	59 (29%)	0.737	4 (13%)	35 (9.4%)	0.954
**Upward melanocytes**	84 (27%)	11 (58%)	**0.036**	11 (79%)	194 (97%)	0.304	29 (91%)	339 (91%)	>0.999
**Pagetoid spread**	8 (2.5%)	2 (11%)	0.279	3 (21%)	99 (49%)	0.404	21 (66%)	263 (71%)	0.954
**Nest confluence**	162 (51%)	12 (63%)	0.703	11 (79%)	116 (58%)	0.555	21 (66%)	278 (75%)	0.954
**Distinct junctional atypia**	29 (9.2%)	4 (21%)	0.279	9 (64%)	160 (80%)	0.555	28 (88%)	343 (92%)	0.954
**Junctional mitoses present**	1 (0.3%)	0 (0%)	>0.999	0 (0%)	30 (15%)	0.566	8 (25%)	119 (32%)	0.954
**Lack of maturation**	14 (4.4%)	1 (5.3%)	0.860	0 (0%)	2 (1.0%)	>0.999	21 (66%)	260 (70%)	0.955
**Distinct dermal atypia**	13 (4.1%)	1 (5.3%)	0.860	0 (0%)	2 (1.0%)	>0.999	20 (63%)	253 (68%)	0.954
**Dermal mitoses present**	0 (0%)	0 (0%)		0 (0%)	0 (0%)		7 (22%)	117 (31%)	0.954
**Ordered single cells (benign pattern)**	204 (65%)	2 (11%)	**<0.001**	3 (21%)	24 (12%)	0.737	2 (6.3%)	26 (7.0%)	>0.999
**Atypical dissociation (malignant pattern)**	30 (9.5%)	2 (11%)	0.934	1 (7.1%)	1 (0.5%)	0.555	18 (56%)	216 (58%)	>0.999
**Regression**	17 (5.4%)	1 (5.3%)	>0.999	2 (14%)	33 (16%)	>0.999	9 (28%)	78 (21%)	0.954
**Dense lymphocytic infiltrate**	56 (18%)	5 (26%)	0.703	5 (36%)	63 (31%)	>0.999	20 (63%)	220 (59%)	>0.999
**Tumor-infiltrating lymphocytes (TILs)**	30 (9.5%)	0 (0%)	0.703	2 (14%)	23 (11%)	>0.999	16 (50%)	166 (45%)	0.954
**Ulceration**	0 (0%)	0 (0%)		0 (0%)	1 (0.5%)	>0.999	2 (6.3%)	43 (12%)	0.954
**Subcutis infiltration**	5 (1.6%)	0 (0%)	>0.999	0 (0%)	0 (0%)		1 (3.1%)	17 (4.6%)	>0.999

[1]Median (Q1, Q3); n (%)

[2]Kruskal-Wallis rank sum test; Fisher's Exact Test for Count Data; Fisher's Exact Test for Count Data with simulated p-value (based on 2000 replicates)
